# Barriers and facilitators to the implementation of the kangaroo mother care in NICUs of high-income and low- and middle-income countries: a scoping review

**DOI:** 10.3389/fpubh.2025.1701738

**Published:** 2026-01-12

**Authors:** Zeyao Shi, Xiaowen Li, Yanling Hu, Ru Yang, Zhaolan Zeng, Shulin Hou

**Affiliations:** 1 Department of Neonatology Nursing, West China Second University Hospital, Sichuan University, Chengdu, China; 2 Key Laboratory of Birth Defects and Related Diseases of Women and Children (Sichuan University), Ministry of Education, Chengdu, China

**Keywords:** kangaroo-mother care method, barriers, facilitators, implementation, NICU

## Abstract

**Aim:**

We aim to conduct an overview of barriers and facilitators to implementing kangaroo mother care (KMC) in neonatal intensive care units (NICUs) across different countries.

**Design:**

This study was guided by Arksey and O’Malley’s framework.

**Data sources:**

We searched the following databases on 15 December 2024: PubMed, Scopus, CINAHL, and Embase. We restricted the searches to articles between 1987 and the search date. The starting year was included because KMC was initiated in 1987.

**Review methods:**

A standardized data extraction form was developed and tested by the team. Data extraction was completed by Author 1 and Author 2. A table was used to extract data and synthesize results.

**Results:**

A total of 1,975 papers were yielded from the database search. We finally included 16 studies for narrative synthesis, executed in five high-income countries (HICs) and 11 in low- and middle-income countries (LMICs): 11 qualitative and four quantitative studies and one mixed-method study. We identified four themes, including hospital factors, parental factors, social-cultural factors, and financial factors, from the included articles. A total of 30 items of barriers and 25 items of facilitators were summarized. Parental lack of KMC knowledge or practice, shortage of medical staff, parental reluctance to participate in KMC, safety concerns on infants of healthcare providers (HCPs), reluctance to allow KMC of HCPs, limited knowledge or practice on KMC of HCPs, social-cultural factors including religious beliefs, gender inequality or lack of women empowerment, and financial factors including no cost-sharing mechanism were all reported only in LMICs. Comfortable facilities, maternity leave, and financial resources were mentioned as facilitators only in HICs. Availability of space or beds, preparation for KMC implementation, localized KMC practice, obliging and competent staff, and recognition of low-birth-weight (LBW) infants were only found as facilitators in LMICs.

**Conclusion:**

Different influencing factors to implement KMC were reported in HICs and LMICs. A better understanding of the key factors can help hospitals and government leadership make related policies to promote KMC practice. We recommend that decision makers design strategies and adopt interventions that specifically address these barriers and facilitators to better uptake of KMC.

**New contribution:**

At present, no studies have identified and synthesized barriers and facilitators of KMC practice, and described different features between HICs and LMICs. In this review, we synthesized barriers and facilitators reported in studies. This scoping review provides government and hospital leaders with evidence to make policies to promote KMC practice.

## Highlights

- There are various characteristics of KMC implementation under different care environments, including improved infants’ survival, vital signs, neurodevelopment, and the quality of parent–child bonding.
- The NICU environment, increased workload of healthcare providers, shortage of staff, lack of KMC knowledge among parents, cultural norms, and lack of funding are unique barriers in LMICs.
- The NICU environment and financial resource support were only reported as facilitators in HICs.
- Limited NICU environments, mothers’ postpartum discomfort and parental safety concerns, and parental lack of time were also reported as barriers in the HICs.

## 1 Introduction

In 2019, more than 2.4 million neonatal deaths (age < 28 days) occurred, accounting for 37.3% of all under-five deaths (1). Preterm birth complications are a primary reason for the death of newborns globally (2). An estimated 13.4 million preterm live births were born in 2020 (3). The highest rate of preterm babies was in India, followed by Pakistan, Nigeria, China, Ethiopia, Bangladesh, Congo, and the USA (3). Although preterm births are more common in low- and middle-income countries (LMICs), rates of 10% or higher have also been reported in high-income countries (HICs) such as the USA and Greece (3). Preterm birth has become a global burden because of the high morbidity and mortality. Therefore, a cost-effective and applicable intervention for preterm newborns in low- to high-income areas is essential.

Kangaroo mother care (KMC) was first described in Colombia in 1987 (4). It is defined as continuous skin-to-skin contact between the infant and the mother’s chest, exclusively breastfeeding, and timely discharge with close follow-up (4, 5). WHO guidelines recommend that short intermittent KMC should be initiated when the baby’s condition begins to be stable, and a full account of continuous KMC sessions should be taken when fully stable (6). KMC is a feasible, safe, and effective intervention. Literature reported a 33 and 23% reduction in low-birth-weight (LBW) infant mortality, respectively, when comparing KMC with conventional care (7, 8). KMC plays an important role in infant survival, vital signs, neurodevelopment, and the quality of parent–child bonding (7, 9, 10). In addition, it facilitates the early initiation of breastfeeding, which effectively decreases the incidence of necrotizing enterocolitis, a significant cause of death in preterm infants (11). Therefore, KMC is highly suggested for scaling across geographies. Several studies have shown that KMC can also be effective in community-based settings, although the WHO specifies that KMC should be initiated in a facility setting (12, 13).

Despite the known benefits, the amount of evidence shows that parents may face multiple-level barriers when providing KMC, some of which prevent them from maintaining continuous skin-to-skin contact with their babies. The literature describes these perceived barriers as experiential, resource-related, sociocultural, and others (14). These perceived barriers may vary from area to area. For example, some deliveries occur at home in LMICs, especially in rural areas. A portion of preterm infants lose the opportunity to receive KMC in the neonatal intensive care units (NICUs) (15). Besides, resource scarcity is another important issue which hampers the KMC. In LMICs, there is not enough medical equipment or staff to meet the conditions for implementing KMC in the NICUs (14). Other difficulties, including lack of knowledge from parents, a localization guide lacking, and traditional beliefs, also hinder the KMC in resource-limited areas (14, 15). KMC also meets some barriers in resource-rich countries, although compared to LMICs, their obstacles are insignificant. For example, a more comfortable reclining chair or a single room was required from parents in HICs. In addition, healthcare providers’ (HCPs) negative attitude and clinical practice variation were also reported as barriers in HICs (16). For facilitators, the availability of space, HCPs’ positive attitude, and other obtained support factors can advance the KMC practice in LMICs. However, few facilitators were reported in HICs, but factors such as financial support and physical environment (16, 17). Different countries face unique obstacles and enabling factors in implementing KMC, and a better understanding of the barriers and facilitators to practice KMC can help implement it more broadly. Previous reports have synthesized the factors influencing the implementation of practicing KMC (14–17). However, there is a dearth of information synthesis on describing the features of these perceived influencing factors in HICs and LMICs. We assume that different countries present unique barriers and facilitators for enabling KMC due to different policies, resources and culture.

## 2 The review

### 2.1 Aims

We aim to conduct an overview of barriers and facilitators to implementing KMC in NICUs in different countries in this study. This review’s question was, ‘What are the factors impeding or enhancing KMC implementation in the NICUs of HICs and LMICs?’

### 2.2 Design

As the available evidence is limited and inconsistent, a scoping review was the most appropriate method to provide an overview of the literature, describe the research being conducted in this area, and highlight areas where more research is needed. This scoping review was guided by five-stage Arksey and O’Malley’s framework (18), including: 1. Identifying the research question; 2. Identifying relevant studies; 3. Study selection; 4. Charting the data; 5. Collating, summarizing, and reporting the results. The study was reported according to the Preferred Reporting Items for Systematic Review and Meta-Analyses, Scoping Review extension (PRISMA-ScR) guidelines (19). The Prisma checklist was provided in the supplementary file. We have registered the protocol of this scoping review on the web of OSF Registries (Registration DOI: https://doi.org/10.17605/OSF.IO/W4QAJ).

### 2.3 Inclusion and/or exclusion criteria

We included studies in this review if they: (i) were published in English; (ii) were full-text articles; (iii) were quantitative, qualitative, or mixed method studies; (iv) reported barriers and/or facilitators to practice KMC in NICU and KMC for preterm infants or LBW infants. Studies were excluded if they: (i) were not written in English; (ii) were grey literature because these grey publications have not undergone peer review; (iii) reported implementation of KMC not in hospital; (iv) reported KMC for term infants; (v) were literature reviews.

### 2.4 Search methods

Four electronic databases were searched: PubMed, Scopus, CINAHL, and Embase, with search dates from the KMC start year of 1987 to 15 December 2024. Table 1 below provides an overview of the search strategy from each database. The search strategy was validated by peer-reviewed.

**Table 1:** Database search strategy.

Date of search	Keyword search	No. of publications retrieved	Database
15 Dec 2024	1. ((skin to skin contact[Title/Abstract]) OR (((((((((((Care Method, Kangaroo-Mother[Title/Abstract]) OR (Care Methods, Kangaroo-Mother[Title/Abstract])) OR (Kangaroo Mother Care Method[Title/Abstract])) OR (Kangaroo-Mother Care Methods[Title/Abstract])) OR (Method, Kangaroo-Mother Care[Title/Abstract])) OR (Methods, Kangaroo-Mother Care[Title/Abstract])) OR (Kangaroo Mother Care[Title/Abstract])) OR (Care, Kangaroo Mother[Title/Abstract])) OR (Kangaroo-Mother Care[Title/Abstract])) OR (Care, Kangaroo-Mother[Title/Abstract])) OR (“Kangaroo-Mother Care Method”[Mesh]))) AND ((((((facilitators[Title/Abstract]) OR (enablers[Title/Abstract])) OR (motivators[Title/Abstract])) OR (experience[Title/Abstract])) OR (perception[Title/Abstract])) OR (attitude[Title/Abstract])) 2. (((((barriers[Title/Abstract]) OR (difficulty[Title/Abstract])) OR (obstruction[Title/Abstract])) OR (obstacle[Title/Abstract])) OR (impediment[Title/Abstract])) OR (setback[Title/Abstract])) AND ((skin to skin contact[Title/Abstract]) OR (((((((((((Care Method, Kangaroo-Mother[Title/Abstract]) OR (Care Methods, Kangaroo-Mother[Title/Abstract])) OR (Kangaroo Mother Care Method[Title/Abstract])) OR (Kangaroo-Mother Care Methods[Title/Abstract])) OR (Method, Kangaroo-Mother Care[Title/Abstract])) OR (Methods, Kangaroo-Mother Care[Title/Abstract])) OR (Kangaroo Mother Care[Title/Abstract])) OR (Care, Kangaroo Mother[Title/Abstract])) OR (Kangaroo-Mother Care[Title/Abstract])) OR (Care, Kangaroo-Mother[Title/Abstract])) OR (“Kangaroo-Mother Care Method”[Mesh]))) 3. 1 AND 2	531	PubMed
15 Dec 2024	1. “Kangaroo care” OR “Kangaroo mother care” OR “Kangaroo mother care method” OR “Skin to skin contact” OR “Skin-skin contact” 2. “facilitators” OR “enablers” OR “motivators” OR 2.”experience” OR “perception” OR “attitude” OR “difficulty” OR “barriers” OR “influencing factors” OR “impediment” 1 AND 2	911	Scopus
15 Dec 2024	#S1: SU (facilitators or enablers or barriers or influencing factors or determinants) OR SU (difficulties or challenges or barriers or issues or struggles or problems or complexities) #S2: SU (kangaroo care or skin-to-skin or kangaroo mother care or skin-to-skin or chest-to-chest or skin contact) OR SU (kangaroo-mother care method or care kangaroo-mother) #S3: #S1 AND #S2	19	CINAHL
15 Dec 2024	#1: ((‘facilitation’/exp. OR ‘facilitation’ OR enables OR influencing) AND factors OR ‘determinants’/exp. OR determinants) AND difficulties OR challenges OR ‘barriers’/exp. OR barriers OR issues OR struggles OR problems OR complexities #2: (‘kangaroo care’/exp. OR ‘kangaroo care’ OR ‘skin to skin contact’/exp. OR ‘skin to skin contact’) #3: #1 AND #2	514	Embase

### 2.5 Review process

**Figure 1:**
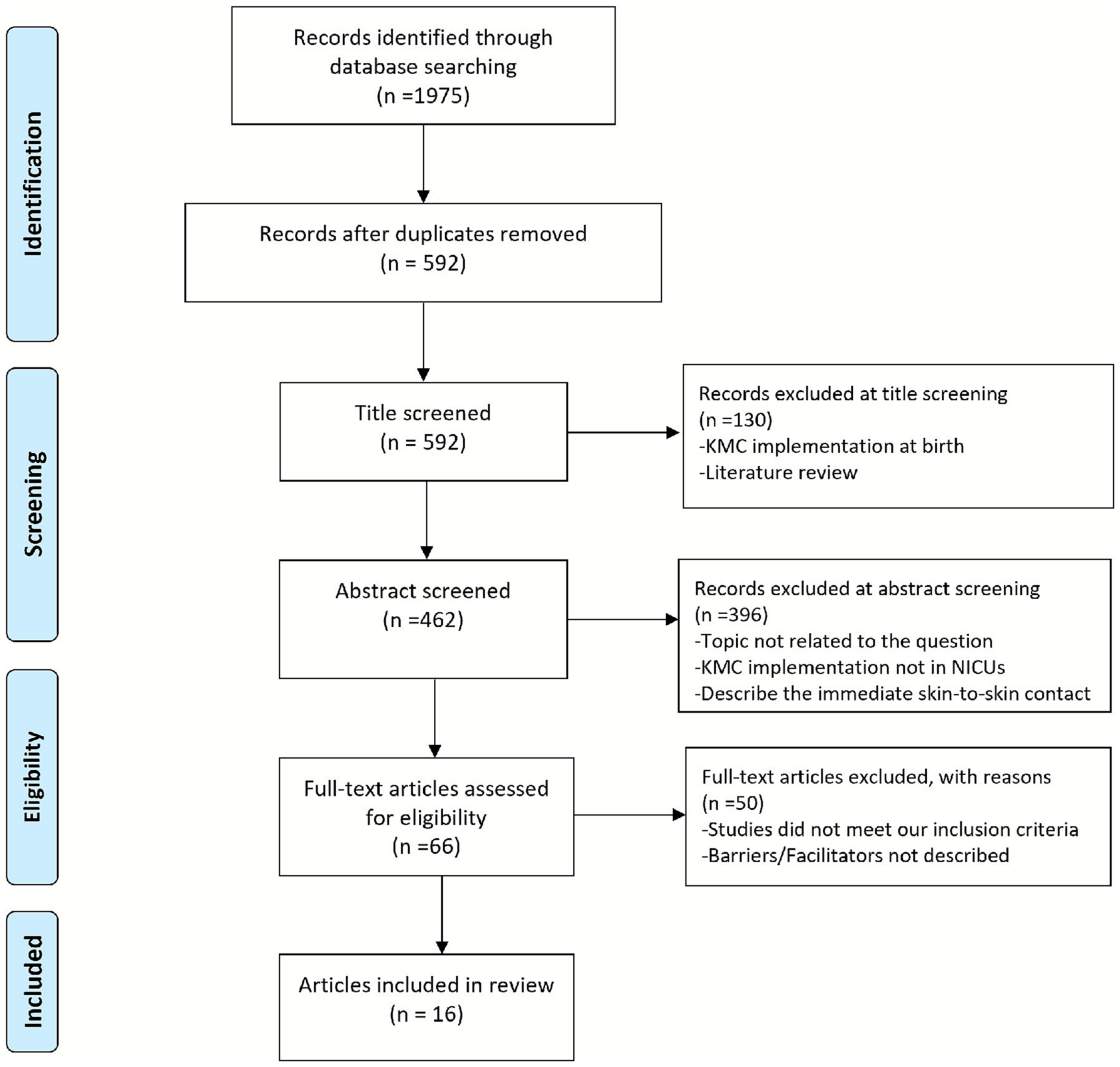
PRISMA scoping review flow diagram.

After 1,383 duplicates were removed, two reviewers (Author 1 and Author 2) first independently screened titles and abstracts according to inclusion and exclusion criteria. A third reviewer (Author 3) resolved any dispute. In this stage, we excluded 526 articles due to the following reasons: (1) KMC was implemented only at birth of immediate skin-to-skin contact, and not in the NICU; (2) Studies were literature reviews; (3) Topic was not related to our question. And then, potentially eligible studies underwent full-text review by the same two reviewers. Studies were excluded if they did not meet the inclusion criteria and did not describe barriers or facilitators, and a total of 50 articles were excluded in this stage. Finally, a PRISMA flow chart was generated (Figure 1).

### 2.6 Data extraction and synthesis

A standardized data extraction form was developed and tested by the team. Data extraction was completed by Author 1 and Author 2. To ensure accuracy, two reviewers discussed discrepancies found in the extracted data. Data items included authors and year, country, study design, study population, study setting, and key findings, including barriers and/or facilitators to implementing KMC. The sum of the research papers identified was categorized utilizing a thematic approach. Barriers and facilitators were divided into hospital, parental, social-cultural, and financial factors. Hospital-related factors included physical environment, medical staff, and clinical practice. Parental factors encompass knowledge, practice, and attitude level of KMC, support from others, and health status. The socio-cultural part comprises beliefs and gender inequality. In addition, financial factors incorporate the financial status of parents or financial support from others. A table was used to extract data and synthesize results.

## 3 Results

### 3.1 Characteristics of included studies

A total of 16 papers were identified, which were published between 2013 and 2024. In terms of design, 11 studies (69%) were qualitative reviews (16, 20–29), and others were quantitative studies (30–33) (*n* = 4) and a mixed method study (34) (*n* = 1). Of these studies, five were conducted in HICs, including Sweden, the USA, Canada, and Italy. A further 11 studies were conducted in LMICs, including China, Malawi, Indonesia, Nigeria, and Uganda. The characteristics of the included studies are summarized in Table 2.

**Table 2:** Characteristics of included studies (*n* = 16).

Author and year	Country	Country classification by income level	Study design	Study setting	Study population
Blomqvist et al. (2013) (33)	Sweden	HIC	Quantitative study	Two NICUs	76 mothers and 74 fathers
Deng et al. (2018) (30)	China	LMIC	Quantitative study	Online	830 neonatal nurses
Lewis et al. (2019) (24)	USA	HIC	Qualitative interviews	Two NICU	20 mothers
Utami et al. (2019) (31)	Indonesia	LMIC	Quantitative study	Four hospitals	111 participants working in NICU and Perinatology wards
Kinshella et al. (2020) (28)	Malawi	LMIC	Qualitative interviews	Four hospitals	27 service providers and supervisors
Mathias et al. (2020) (26)	Malawi	LMIC	Qualitative interviews	One hospital	Six LBW infants’ parents and six high-risk pregnant mothers
Yue et al. (2020) (20)	China	LMIC	Qualitative interviews	Five hospitals	38 health providers and parents
Coutts et al. (2021) (16)	Canada	HIC	Qualitative interviews	11NICUs	35 healthcare providers
Artese et al. (2021) (22)	Italia	HIC	Qualitative interviews	86 NICUs	107 NICU directors
Bilal et al. (2021) (23)	Ethiopia	LMIC	Qualitative interviews	30 health centers and 85 health posts	144 health service providers and community members
Asmare et al. (2021) (27)	Ethiopia	LMIC	Qualitative interviews	One hospital	13 mothers and 7 nurses
Saltzmann et al. (2022) (34)	USA	HIC	Mixed-methods study	One NICU	50 parents
Esewe et al. (2022) (25)	Nigeria	LMIC	Qualitative interviews	One NICU	13 mothers
Wang et al. (2023) (21)	China	LMIC	Qualitative interviews	Four health facilities	155 participants, including stakeholders of the Safe Neonatal Project
Utami et al. (2023) (32)	Indonesia	LMIC	Quantitative study	Four hospitals	86 parents and family members
Tumukunde et al. (2024) (29)	Uganda	LMIC	Quantitative study	Four hospitals	23 healthcare workers and 41 caregivers

### 3.2 Synthesis of results

Finally, barriers and facilitators were divided into hospital, parental, social-cultural, and financial factors. A total of 30 items of barriers and 25 items of facilitators were summarized, respectively, across 16 studies. The summary of barriers and facilitators to KMC implementation is outlined in Tables 3, 4.

**Table 3:** Summary of barriers to KMC implementation.

Barriers influencing KMC implementation		References	Description
Hospital factors			
The NICU physical environment	Limited facilities or medical equipment	(21–23, 25, 28, 32–34)	4 HICs and 4 LMICs
	Lack of space or privacy	(16, 20, 25, 29, 30, 32–34)	4 HICs and 4 LMICs
HCP-related factors	Doubtful beliefs	(16)	1 HIC
	Limited communication and messaging to parents	(16, 28)	1 HIC and 1 LMIC
	Increased workload	(16, 20, 29, 32)	2 HICs and 2 LMICs
	Shortage of staff	(20, 25, 28)	3 LMICs
	Safety concerns on infants	(20, 29–31)	4 LMICs
	Limited knowledge or practice on KMC	(21, 30)	2 LMICs
	Reluctance to allow KMC	(29–31)	3 LMICs
	Negative nurses’ attitudes	(25, 32–34)	2 HICs and 2LMICs
	Lack of leadership involvement	(29)	1 LMIC
Clinical practice-related factors	Lack of KMC policies or guidelines	(16, 21)	1 HIC and 1 LMIC
	Restrictive parent access policy	(21, 22)	1 HIC and 1 LMIC
Parental factors			
Knowledge, practice, attitude	Lack of KMC knowledge or practice	(21, 23, 25, 26, 29, 30)	6 LMICs
Obtained support	Lack of fathers’ involvement	(21)	1 LMIC
	Gossip and ridicule from friends	(25)	1 LMIC
	Discouragement from others	(25)	1 LMIC
	Reluctance to participate in KMC	(20, 29, 30)	3 LMICs
	Lack of time	(30, 32–34)	2 HICs and 2 LMICs
Physical and mental health	Maternal stress or anxiety associated with preterm birth	(20, 24)	1 HIC and1LMIC
	Postpartum discomfort	(21, 24, 27, 29, 32, 33)	3 HICs and 3LMICs
	Safety concerns on infants	(20, 24, 27, 29, 32, 33)	3 HICs and 4 LMICs
Social-cultural factors			
Postpartum confinement culture		(20)	1 LMIC
Delivery at home		(23)	1 LMIC
Religious beliefs		(23, 26)	2 LMICs
Gender inequality or lack of women’s empowerment		(23, 26)	2 LMICs
Financial factors			
Lack of dedicated funding or financial resources		(21, 25)	2 LMICs
Health insurance		(21)	1 LMIC
No cost-sharing mechanism		(21, 29)	2 LMICs
Poor referral system		(23)	1 LMIC

**Table 4:** Summary of facilitators to KMC implementation.

Facilitators influencing KMC implementation		References	Description
Hospital factors			
The NICU physical environment	Comfortable facilities	(22, 32)	2 HICs
	The availability of space or beds	(29)	1 LMIC
HCP-related factors	Positive attitude	(21, 27, 28)	3 LMICs
	Pre-preparing for KMC implementation	(21)	1 LMIC
	Trained or skilled KMC providers	(26)	1 LMIC
	Wealthy knowledge	(26)	1 LMIC
	Obliging and competent staff	(32)	1 LMIC
Clinical practice-related factors	Daily repeated KMC sessions and KMC annotation	(22)	1 HIC
	Successfully implemented in some of the pilot counties	(21)	1 LMIC
	Incorporated KMC into the clinical process	(21)	1 LMIC
	Localized KMC practice	(21)	1 LMIC
	The availability of medicine and timely treatment	(29)	1 LMIC
Parental factors			
Knowledge, practice, attitude	Conviction of KMC’s advantage	(26, 32)	1 HIC and 1 LMIC
	Parental affection	(26)	1 LMIC
	Recognition of LBW infants	(26)	1 LMIC
	The positive experience of KMC	(26)	1 LMIC
Obtained support	Family support and involvement	(27, 29, 32)	1 HIC and 2 LMICs
	Maternity leave	(24)	1 HIC
	Cultural and religious support	(27)	1 LMIC
	Support from the leadership	(20, 21)	2 LMICs
	Peer support education	(26)	1 LMIC
	Community support	(20, 26, 29)	3 LMICs
	Health linkage system	(26)	1 LMIC
Financial factors			
Parents with good financial status		(20)	1 LMIC
Financial resources		(24)	1 HIC

### 3.3 Factors influencing the implementation of KMC in the NICUs

#### 3.3.1 Barriers

A total of 30 barriers were extracted across 16 studies. Twelve and 24 items of barriers were found in HICs and LMICs, respectively. NICU environmentally limited facilities or medical equipment (21–23, 25, 28, 32–34) and lack of space or privacy (16, 20, 25, 29, 30, 32–34) were the most common barriers in both HICs and LMICs reported by eight studies, respectively. Seven studies described parental concern about infants’ safety (20, 24, 27, 29, 32, 33) during KMC practice as a barrier in both HICs and LMICs. Postpartum discomfort (21, 24, 27, 29, 32, 33)^,^ such as backache, was mentioned in both HICs and LMICs, which was reported by six studies. Increased workload (16, 20, 29, 32), negative nurses’ attitude (25, 32–34), and parental lack of time (30, 32–34) were discussed in four studies, respectively, which were both common in HICs and LMICs. Parental lack of KMC knowledge or practice was only found as a barrier in six studies (21, 23, 25, 26, 29, 30) in LMICs. Other barriers, such as shortage of medical staff (20, 25, 28), parental reluctance to participate in KMC (20, 29, 30), safety concerns on infants of HCPs (20, 29–31), reluctance to allow KMC of HCPs (29–31), limited knowledge or practice on KMC of HCPs (21, 30), social-cultural factors including religious beliefs (23, 26), gender inequality or lack of women empowerment (23, 26), and financial factors including no cost-sharing mechanism (21, 29) were all reported only in LMICs. HCPs’ doubtful belief in the perceived value of KMC practice was thought of as a barrier only in one HIC (16).

#### 3.3.2 Facilitators

Twenty-five items of facilitators were found in nine studies in total. Seven and 21 items of facilitators were found in HICs and LMICs, respectively. HCPs’ positive attitude toward the benefits of KMC (21, 27, 28), family support and involvement (27, 29, 32), and community support (20, 26, 29) were the most common facilitators reported by three literatures, respectively. Two studies reported parental conviction of KMC’s advantage (26, 32) as a facilitator in both HICs and LMICs. Two studies discussed comfortable facilities (22, 32) as an enabling factor only in HICs. Support from the leadership (20, 21) was mentioned as a facilitator only in two LMICs’ studies. Other facilitators, including daily repeated KMC sessions and KMC annotation on infants’ medical records (22), maternity leave (24), and financial resources (24) were mentioned only in HICs. Some items such as the availability of space or beds (29), preparation for KMC implementation (21), localized KMC practice (21), obliging and competent staff (32), and recognition of LBW infants (26) were only found in LMICs.

## 4 Discussion

This scoping review aimed to identify the barriers and facilitators of KMC implementation in the NICUs of HICs and LMICs. Furthermore, we sought to synthesize and describe the different characteristics between HICs and LMICs. In this review, we have identified four themes that include hospital factors, parental factors, social-cultural factors, and financial factors. Finally, we concluded 30 items of barriers and 25 items of facilitators.

### 4.1 Common barriers in both HICs and LMICs

Firstly, the NICU physical environment, including limited facilities or medical equipment and a lack of space or privacy, was the most frequent barrier. Literature in both LMICs and HICs reported limited NICU environments, such as beds, chairs, wraps, and private space, in influence KMC practice heavily. Studies showed that equipment was one of the key factors affecting KMC practice (17, 35, 36). In resource-limited areas such as China, a populous country with more than 1.4 billion people and 9.02 million births in 2023 (37), tertiary-level hospitals constantly struggle to find enough space for NICUs due to the population density and large numbers of births. Accommodating space, comfortable chairs, and sufficient privacy for parents providing KMC in NICUs is an additional burden (20). Most NICUs in LMICs are open-bay spaces, with incubators, monitors, and ventilators crowded in the wards. It is difficult to provide basic facilities such as chairs, beds, and curtains for KMC practice. Besides, a lack of privacy was another reported problem. Most mothers reported feeling uncomfortable with exposed breasts during KMC provision, as medical staff continued to come and go in open spaces (38). However, in resource-supported settings like Canada, some NICUs are designed as single-family rooms or semi-private room designs, including a bed or couch for parents. In addition, continuous rather than intermittent KMC was practiced in HICs. The barriers most reported were limited space for reclining chairs beside each infant incubator or crib in the open-bay facility and the absence of individual rooms with a bathroom, as some families will be here 24/7 (16).

Secondly, both LMICs and HICs reported mothers’ postpartum discomfort and parental safety concerns as obstacles to KMC. Except for ongoing complex emotions after sudden birth, most mothers had to endure physical discomfort such as back and stomach pain (24). Physical recovery limited self-care and influenced mothers’ ability to devote energy to their children. Besides, many mothers had anxiety about carrying for preterm infants. On one hand, they were afraid of hurting the babies while performing skin-to-skin contact. On the other hand, parents became more hesitant to perform KMC as the infants’ vital signs changed (20, 24, 27, 29)_._

Thirdly, studies in both HICs and LMICs reported parental lack of time as a difficulty in KMC practice. Several parents expressed that they cannot balance time between family and practicing KMC in the NICU (33). Except for caring for preterm babies, they may have to accompany older siblings at home and handle the household. Moreover, negative nurses’ attitudes also hindered KMC practice. Parents defined staff support as the provision of three key components namely; positive encouragement, information, and practical assistance (33). However, several parents reported that some staff did not help to position infants on a parent’s chest, and parents were sometimes disturbed by staff when performing KMC (32–34). Besides, some staff hold negative perceptions of KMC (33). The staff’s negative attitude and behaviors were contrary to the parents’ strong desire of being close to their infants.

### 4.2 Unique barriers in LMICs

Firstly, only studies conducted in LMICs discussed the lack of knowledge among parents influencing the carry-out of KMC. A research study (23) in southern Ethiopia showed that mothers and other families refused to receive KMC because they considered that preterm and LBW infants do not survive and grow like normal babies. The lack of information and awareness about preterm infants leads to poor uptake of KMC practice. Besides, another study (38) reported that parents were simply told to practice KMC without explaining why and how to do it. They felt forced to do it, as such the mothers were less likely to accept KMC. This phenomenon may be due to poor counseling or a lack of counseling materials from medical staff. In addition, KMC was considered the “poor man’s alternative,” a sub-standard method of care in some developing countries (39). The under-recognition of KMC hindered the progress of KMC practice. In contrast to LMICs, KMC was more widely spread and advocated in HICs; parents may have an affluent knowledge of KMC and preterm or LBW infants.

In addition, increased workload and shortage of staff were more commonly reported in LMICs. Nurses, doctors, or allied health providers who render KMC service are an important factor. A study conducted in Nigeria showed that one nurse should look after more than eight babies in a shift (25). The shortage of pediatric doctors and nurses is a major concern in China as well. Compared with developed countries, China is short of at least 200,000 pediatricians (40), and the nurse-to-patient ratio is lower than in HICs, such as in the United States, where the average nurse cares for two infants (41). Training and supervising mothers to practice KMC and recording and collecting KMC data can be a further burden for medical workers (38).

It is noteworthy that HCPs’ safety concerns were only discussed in LMICs, especially in China. The majority of NICUs in China are restrictive for parents’ visits (42). The closed-off management policy originated from the hospital’s concern about nosocomial infection. Due to the culture of postpartum confinement, many mothers do not shower. Besides, the NICUs are often overcrowded. Medical staff expressed huge concern about KMC and feared that it may lead nosocomial infection (20). However, family-centered care has been widely promoted in the NICUs of HICs (43). Parent’s presence in the NICU is usual. Other countries, such as Uganda and Indonesia, reported HCPs’ fear of accidental extubation, and parents may harm the unstable infants during KMC (31). In fact, the fear was associated with the staff’s lack of KMC training and the shortage of human resources.

We found that all socio-cultural-related barriers were discussed only in LMICs, especially in Africa. In Ethiopia (23) and Malawi (26), family members felt grief and distress when the mother gave birth to a preterm or LBW infant, and as they believed that the birth was a curse. Besides, they associated LBW delivery with committing a cultural taboo of abortion. The cultural and traditional beliefs hindered the recognition of KMC and subsequently led to non-acceptability and non-utilization. Secondly, male dominance also hampered KMC initiation. In some lagging-behind areas, mothers lack women’s empowerment in making the decision to accept and utilize the KMC service (26). Most mothers depend on their husbands to decide on performing KMC. This gender inequality influenced decision-making in seeking health services. However, females in HICs may have a higher social status, and women are treated with more tolerance and respect. Besides, they may not associate preterm birth with religion. Finally, the lack of financial support was detrimental to KMC implementation. In some LMICs, KMC was not included in the scope of health insurance as it was not a billable item under government regulation (21). Though KMC is a low-cost intervention, it still needs investments in consumables or equipment, staffing, and technology support in provisioning basic needs. These costs were all borne by the hospitals themselves. Lack of funding is a major challenge to KMC in developing countries.

### 4.3 Common facilitators in HICs and LMICs

Family support and involvement (27, 29, 32) were a common enabler, both reported in one HIC and two LMICs. Family support was described as family members assisting with the household and encouraging and motivating mothers to perform KMC (27). If mothers are busy with heavy household chores, they may have difficulty performing KMC in the NICU. A study conducted in Sweden showed (33) that looking after other older children and performing household work hampered parents from spending more time in the NICU with their preterm infants.

Besides, parents’ conviction of KMC’s advantage (26, 32) was reported as a facilitator in both HIC and LMIC. Most parents perceived KMC as safe to utilize, and some of them preferred KMC to incubator care in these studies (26, 33). On the one hand, parental affection towards preterm and LBW infants enables them to accept and utilize KMC (44). On the other hand, some mothers had prior knowledge of KMC through peers or media, enhancing them to seek KMC services (26).

### 4.4 Unique facilitators in HICs

Two reports in Sweden (33) and Italy (22) regarded the comfortable facilities, such as armchairs, height-adjustable beds, and wireless and portable monitoring equipment, as an advantage during the process of KMC. In HICs, the existing infrastructure was already fully capable of meeting the basic needs of caring for KMC. Parents had a greater desire for comfortable and convenient facilities when they performed KMC. Due to the different financial status, the basic facility was still a key barrier in LMICs. However, a study conducted in Italy (22) proved that single-family rooms do not influence the implementation of KMC when compared with open-bay. Evidence suggested that parents perceived higher levels of stress and isolation in single-family units in the NICU. We may conclude that single-family rooms are optional in NICU, though it is helpful to parents’ privacy. Finally, financial support was reported as an enabling factor in the United States (24). Social workers provided parents with financial resources such as parking vouchers and gas cards to ease the burden of transportation. However, a lack of financial support was a dominant obstacle in LMICs. Parents in LMICs have to pay for themselves in accommodation and transportation. Due to these unaffordable costs, parents with poor financial status may disrupt the practice of KMC.

### 4.5 Unique facilitators in LMICs

The medical staff’s positive attitude toward KMC was discussed as the most common facilitator in resource-limited countries. Studies in Ethiopia (27) and India (28) reported the perception of health workers about the benefits of KMC, promoting the utilization of KMC. The detailed information on KMC benefits given by HCPs can improve the willingness of mothers. In LMICs, parents reported a lack of KMC knowledge; they felt encouraged and motivated when HCPs were positive and obliging in the process of KMC.

Besides, the results showed that support from the community (20, 26, 29), leadership (20, 21), and peer education (26) were facilitators for parents to conduct KMC. Literature showed that if HCPs provide a supportive community, such as a social media group, parents can understand more benefits of KMC and share their KMC experience (20). Leadership from hospital management and the government was essential to the KMC’s development. A study reported that leadership and governance were rated as having significant or very major bottlenecks. KMC lacks priorities and institutionalization by regulatory bodies in Asia and Africa (45).

Through this study, we thought that integrating the facilitators and barriers of KMC with an implementation science framework, such as the Consolidated Framework for Implementation Research (CFIR), can transforms the promotion of this evidence-based practice into a structured and strategic process. This systematic approach moves beyond simply identifying challenges like staff shortages or parental anxiety and allows for a comprehensive diagnosis by mapping these factors onto core domains like intervention characteristics, inner and outer settings, individual involved, and the implementation process itself. The key value of this integration lies in its ability to translate identified barriers directly into targeted, actionable implementation strategies.

## 5 Conclusion

KMC is an effective intervention. However, there are different challenges and enabling factors in implementing KMC in HICs and LMICs. According to the results, we came up with some suggestions in order to help promote KMC practice. For example, policies from countries and hospitals should be launched, especially in the LMICs. Firstly, governments and hospitals are responsible for KMC dissemination. Systematic training at the national level is needed to improve the competence of medical staff. Through this approach, parents may receive more support and knowledge of KMC, and they will practice KMC more proactively. Meanwhile, HCPs may eliminate the negative attitude toward KMC after they are trained in KMC-related knowledge. In addition, the state may establish key indicators as well: for example, tracking the proportion of infants weighing less than 2,000 grams who receive KMC daily, or incorporating KMC into national clinical guidelines, to promote its implementation in the hospitals. In addition, compared to HICs, the shortage of staff should be resolved in the LMICs. Hospital leaders can assign dedicated staff to KMC. Sufficient staff not only settle the problem of helping parents to implement KMC, but also can ensure the safety of infants during KMC. Thirdly, socio-cultural-related barriers are deeply rooted in some LMICs. The government should empower women with equal education and employment opportunities, and promote gender equality. Also bear in mind the socio-cultural factors stated above. Men should also be educated about KMC and its benefits. Finally, government and hospital leaders should invest appropriate funds to supply necessary facilities to support KMC. Only the physical environment is improved; the quality of KMC will be better and spread.

## 6 Limitation

Firstly, we did not conduct a supplementary hand search because of resource and time constrain. Secondly, we only reported barriers and facilitators of KMC conducted in the NICUs. Thirdly, although we summarized barriers and facilitators from the literature, there may be a bias or potential data imbalance because the selected studies were mostly from LMICs. Again, the data imbalance may result in over-representation of LMIC studies, which might skew synthesis toward resource-limited contexts. Finally, although our results showed multiple barriers and facilitators, the discussion section prioritizes only key factors due to space constraints, potentially leaving other findings less explored.
